# Evaluation of changes in salivary composition in renal failure patients before and after hemodialysis

**DOI:** 10.4317/jced.54027

**Published:** 2017-11-01

**Authors:** Nishath Khanum, Mahesh Mysore-Shivalingu, Srisha Basappa, Archana Patil, Santosh Kanwar

**Affiliations:** 1 Senior Lecturer, Farooqia Dental College and Hospital, Mysore; 2 Reader, Farooqia Dental College and Hospital, Mysore; 3 Professor and Head, Farooqia Dental College and Hospital, Mysore

## Abstract

**Background:**

Saliva plays a major role in preserving the integrity of oral tissues. The oral health of renal failure patients could be negatively affected by the underlying pathology, the dialysis treatment, or an altered salivary composition. Major systemic changes occur during hemodialysis (HD), which could affect the flow rate and biochemical composition of saliva. Therefore, the aim of this study was to evaluate the effects of HD on the salivary flow rate, pH and biochemical composition before and after completion of a dialysis session.

**Material and Methods:**

Thirty Renal failure patients undergoing hemodialysis were selected based on the inclusion and exclusion criteria set forth for the study. Unstimulated whole saliva (UWS) was collected by the spitting method, immediately before and after a dialysis session. Salivary flow rate, pH, concentration of urea, creatinine, sodium, chloride, potassium and calcium were measured.

**Results:**

Hemodialysis had an acute stimulating effect on the salivary flow rate. The mean pH of UWS showed no significant changes before and after dialysis. The concentrations of urea, creatinine, chloride and potassium in whole saliva changed markedly before and after a hemodialysis session; whereas no significant difference was seen in the concentration of sodium and calcium.

**Conclusions:**

This study shows that HD has significant acute effects on both salivary secretion and biochemical composition in saliva. We conclude that the observed changes in salivary concentrations and flow rate are mainly due to an increased watery secretion from the salivary glands and also saliva can be used as a tool for monitoring hemodialysis.

** Key words:**Saliva, Hemodialysis, Flow rate, pH, biochemical constituents.

## Introduction

Saliva is the body’s mirror. The ability to use saliva to monitor the health and disease state of an individual is a highly desirable goal for health promotion and health care research. Molecular diagnostics contributes into a wide range of disciplines including drug development, personalized medicine (pharmacogenomics) and plays a major role in discovery of biomarkers for the diagnosis of oral and systemic diseases. In the present study we focus on the use of salivary biomarkers for the diagnosis of End Stage Renal Disease (ESRD) and also for determining the effectiveness of hemodialysis.

Over the last decades, the prevalence and incidence of ESRD has increased. The incidence increases with age and male individuals are more commonly affected than females. The most common causes of ESRD are chronic hypertension, glomerulonephritis, polycystic kidney disease, renovascular disease and diabetes mellitus (1,2).

Patients with ESRD can rely on kidney replacement therapeutic modalities such as hemodialysis (HD), peritoneal dialysis (PD) or renal transplantation. Although kidney replacement therapies have proven to be successful in prolonging the life expectancy of ESRD patients, several limitations and long-term complications exist (3). Dialysis leads to systemic alterations, oral complications and variations in the flow and composition of saliva. Salivary function including lubrication, buffering action, maintenance of tooth integrity, antibacterial activity, taste and digestion may be disturbed by salivary flow and biochemical alterations (4). In ESRD patients, the oral health could also negatively be affected by the underlying pathology, the dialysis treatment, oral dryness or an altered salivary composition (5,6).

Measurement of biological markers that demonstrate identifiable and regular changes from pre dialysis to post dialysis states can enable necessary monitoring of dialysis efficacy and the level of renal function in patients with end stage renal disease. The present study was undertaken to analyze the flow rate, pH and biochemical composition (urea, creatinine, sodium, chloride, potassium, calcium) of saliva in patients with renal failure undergoing hemodialysis. This study helps us to determine the salivary biomarkers to monitor dialysis efficacy and also to understand the effects of hemodialysis on salivary composition, function and the relationship between oral and salivary changes in renal insufficiency.

## Material and Methods

The present study was carried out by the Department of Oral Medicine and Radiology, Farooqia Dental College and Hospital, Mysuru to determine the effect of hemodialysis on the flow rate, pH and biochemical composition of saliva (urea, creatinine, sodium, chloride, potassium, calcium) in chronic renal failure patients before and after a hemodialysis session.

Ethical clearance was obtained from the institutional ethical committee to perform this study.

The study sample comprised of thirty subjects with ESRD, who were selected from the outpatients undergoing hemodialysis in the Department of General Medicine, Krishna Rajendra Hospital, Mysuru by simple random sampling based on the following inclusion and exclusion criteria.

Inclusion criteria:

Patients of either sex in the age group of 18 – 80 years, with End stage renal disease having a glomerular filtration rate of < 15 ml/min, undergoing hemodialysis were included in the study.

Exclusion criteria:

Patients with any of the following features were not included in the study:

•Diseases affecting water and electrolyte balance like Diabetes Insipidus, Thyroid disorders except Diabetes Mellitus.

•Patients under medication (other than insulin and antihypertensives) that could affect saliva production.

•Patients with salivary gland disorders.

Patients were explained about the procedure and after obtaining the informed consent unstimulated whole saliva was collected by the spitting method. Saliva was collected immediately before a dialysis session (before) and directly after completion of the dialysis session (after).

For estimation of salivary flow rate, all subjects were instructed to refrain from eating and drinking for one hour prior to the saliva collection. In each patient, the samples were collected during one dialysis session. The collection started with the instruction to void the mouth of saliva by swallowing. Subsequently, saliva was allowed to accumulate in the floor of the mouth and the subjects were instructed to spit out into the sterile calibrated container every 30 seconds. Each saliva collection period was five minutes and following sample collection the flow rate (ml/min) was calculated.

After saliva collection, pH was measured using the pH indicator strip (Merck). One drop of the collected saliva sample was placed on the test strip and its color change indicated the pH of saliva.

The samples were stored in an ice box at -20 degree Celsius and sent to laboratory within 30 minutes of sample collection. The saliva samples were then centrifuged at 4000 rpm for 10 minutes, to eliminate cellular debris. Then the concentration of salivary urea, creatinine, sodium, chloride, potassium and calcium were determined using auto analyzer.

The results were subjected to statistical analysis using Descriptive statistics, Chi-square test, Paired sample‘t’ test and Contingency coefficient analysis. The statistical analysis was done using the SPSS version 16 statistical software package. Data were collected, tabulated and then subjected to the statistical analysis. The qualitative data were presented as numbers and percentages and the Chi square test was used to examine the significance of the differences in mean and distribution of categorical variables between groups. The data were presented as mean ± standard deviation and the paired sample t-test was used for comparison of values before and after hemodialysis.

## Results

Salivary flow rate:

To compare the salivary flow rate before and after hemodialysis paired samples statistics was applied. The mean flow rate before was 0.46 ± 0.27 mL/min and mean flow rate after hemodialysis was 0.84 ± 0.34mL/min. There was a statistically significant difference between the salivary flow rate before and after hemodialysis, as *P* value of 0.000 was obtained.

Salivary pH:

The mean pH before hemodialysis was 6.39 ±1.1 and mean pH after was 6.54 ± 0.60. There was no statistically significant difference between the salivary pH before and after hemodialysis, as the *P* value was 0.239.

Saliva composition:

Biochemical parameters measured in saliva are shown in  Table 1.

**Table 1:**
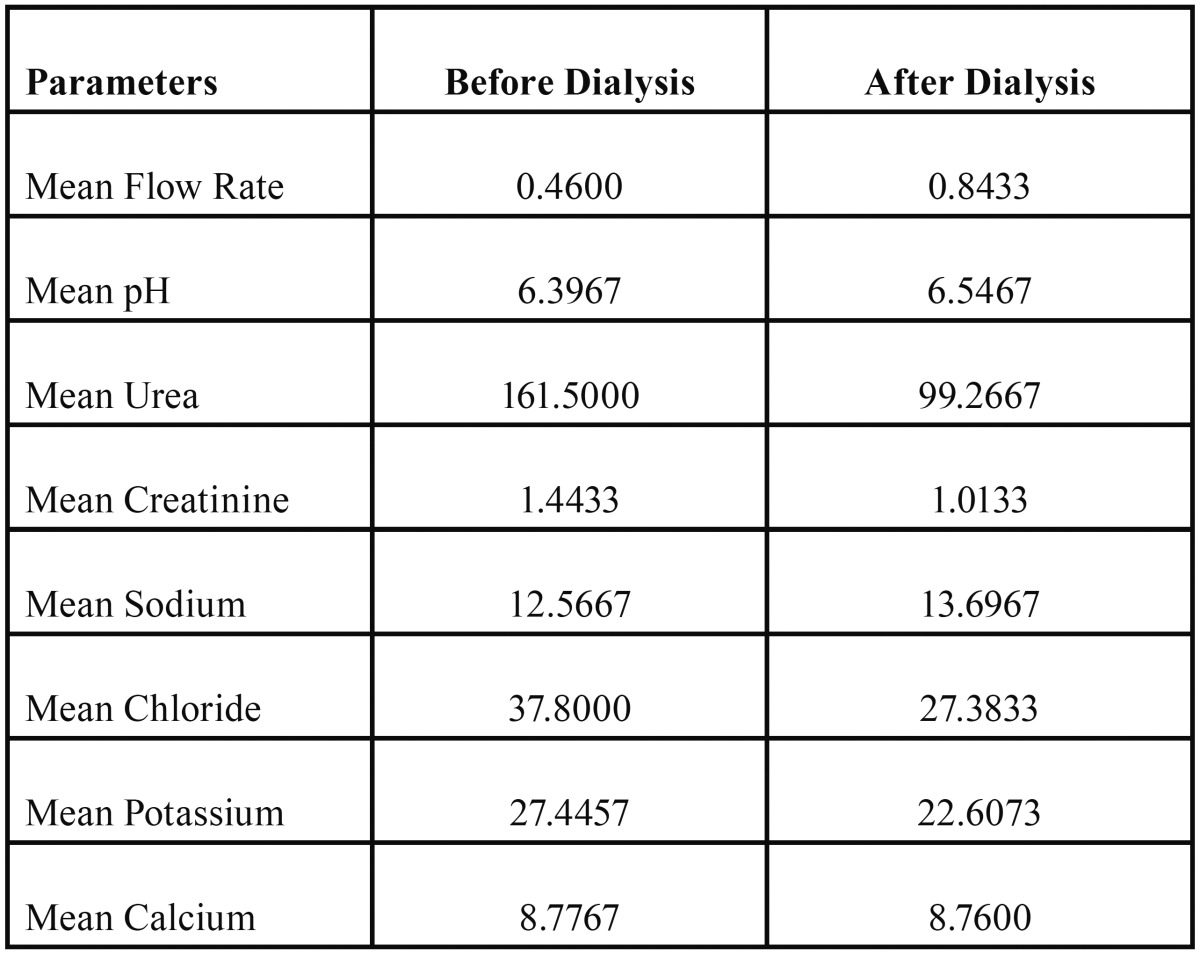
Shows the mean concentration of the salivary flow rate in ml/min, pH and biochemical constituents Urea in mg/dl, Creatinine in mg/dl, Sodium in mmol/L, Chloride in mmol/L, Potassium in mmol/L, Calcium in mg/dl, before and after hemodialysis.

The salivary composition of Chronic Kidney Disease (CKD) patients differed before and after a dialysis session. Concentrations of urea, creatinine, potassium and chloride were found to be significantly lower in patients after hemodialysis than it was before hemodialysis. There were no statistically significant changes seen in the concentrations of sodium and calcium, before and after hemodialysis.

The mean concentration of urea before hemodialysis was 161.50 ± 90.21 mg/dl and mean urea concentration after was 99.26 ± 62.87 mg/dl, with a *P* value of 0.000 which is statistically significant. The mean concentration of creatinine before was 1.4 ± 0.6 mg/dl and mean creatinine concentration after was 1.01 ± 0.56 mg/dl, which was also statistically significant.

The mean sodium concentration before hemodialysis was 12.56 ± 8.07 mmol/L and mean sodium concentration after was 13.69 ± 6.95 mmol/L; The mean calcium concentration before hemodialysis was 8.77 ± 0.53 mg/dl and mean calcium concentration after was 8.76 ± 0.52 mg/dl which was not significant statistically with a *P* value of 0.114 and 0.876 respectively ( Table 2).

**Table 2:**
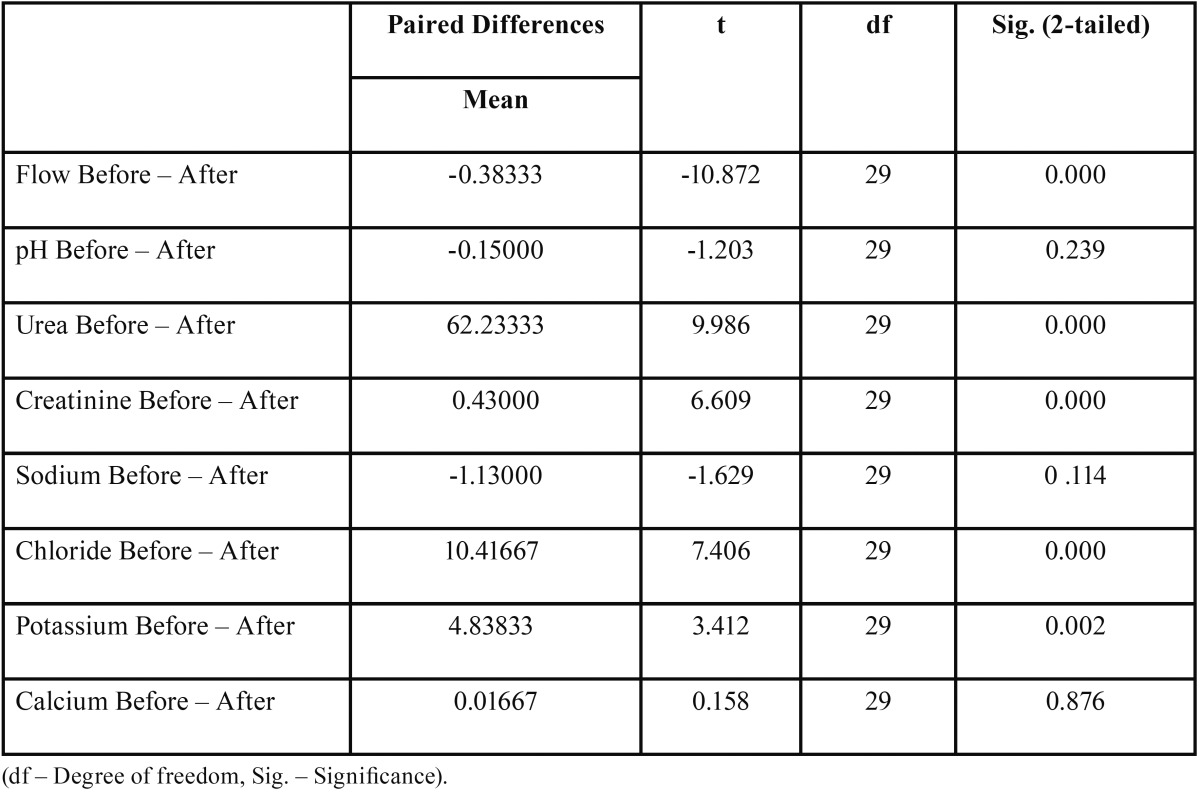
Shows the Paired Samples t-test of difference in salivary constituents before and after hemodialysis.

**Figure 1:**
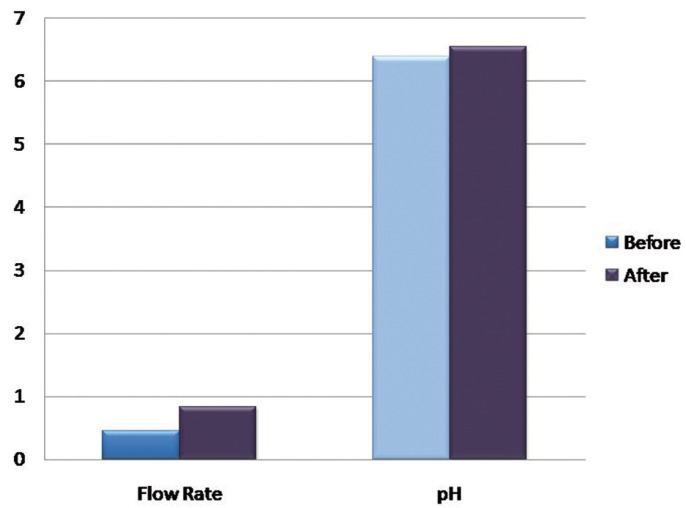
Graph showing the mean Salivary flow rate in ml/min and pH before and after hemodialysis.

**Figure 2:**
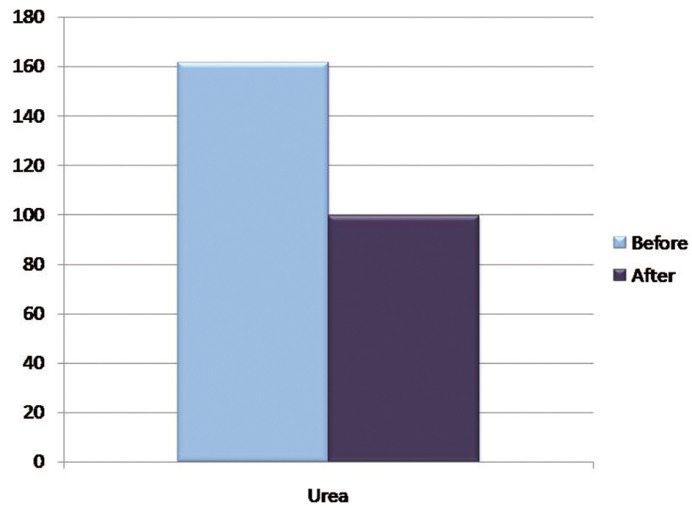
Graph showing the mean concentration of Salivary Urea before and after hemodialysis in mg/dl.

**Figure 3:**
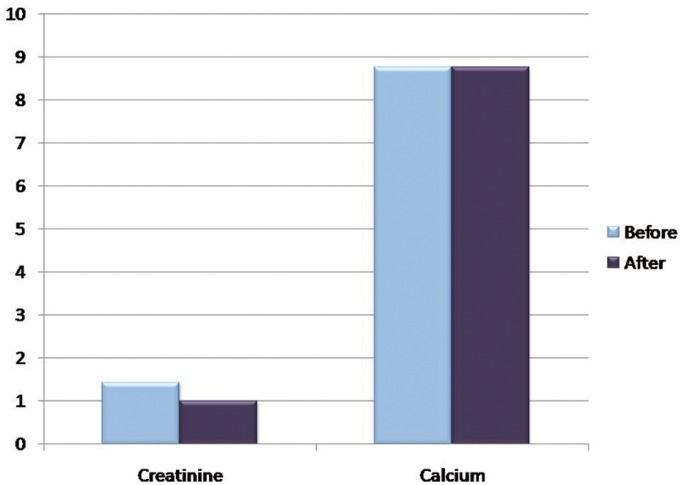
Graph showing the mean concentrations of Salivary Creatinine and Calcium before and after hemodialysis in mg/dl.

**Figure 4:**
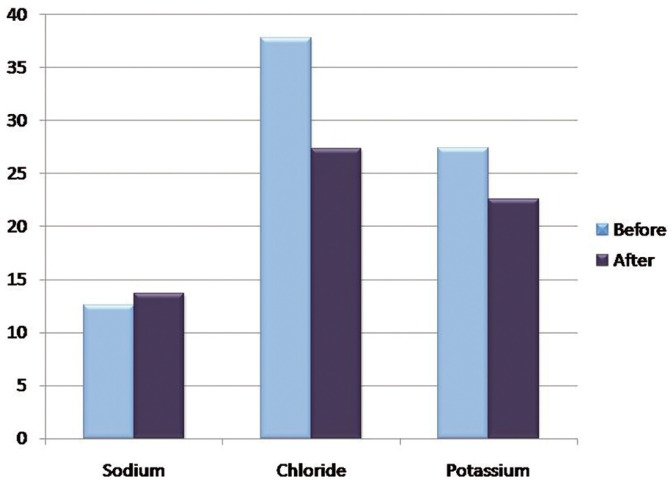
Graph showing the mean concentrations of Salivary Sodium, Chloride and Potassium before and after hemodialysis in mmol/L.

The mean concentration of chloride before hemodialysis was 37.80 ± 13.63 mmol/L and mean chloride concentration after was 27.38 ± 11.85 mmol/L; The mean potassium concentration before hemodialysis was 27.44 ± 9.51 mmol/L and mean potassium concentration after was 22.60 ± 7.58 mmol/L which was statistically significant with a *P* value of 0.000 and 0.002 respectively ( Table 2), (Figs. 1-4).

## Discussion

The ability to monitor health status, disease onset, progression, and treatment outcome through non-invasive means is a highly desirable goal in health care promotion and delivery. Saliva is a perfect medium to be explored for health and disease surveillance. The advantages of saliva as a clinical tool over serum and tissues are a noninvasive collection of sample, smaller sample fractions, good patient cooperation, cost effectiveness, easy storage and transportation, repeated sampling for monitoring over time, greater sensitivity, and correlation with levels in blood.

Chronic renal failure (CRF) is characterized by progressive loss of kidney function. Patients with CRF usually have some oral manifestations of the disease, and one way to improve quality of life is to promote oral health. Oral manifestations generally observed in patients with CRF include enamel hypoplasia, pallor of the oral mucosa, xerostomia, uremic odor, low caries prevalence in both primary and permanent dentitions, as well as a large amount of calculus (6).

Although kidney replacement therapies have proven to be successful in prolonging the life expectancy of ESRD patients, several limitations and long-term complications exist (7). Dialysis leads to systemic alterations, oral complications and variations in the flow and composition of saliva. Salivary functions including lubrication, buffering action, maintenance of tooth integrity, antibacterial activity, and taste and digestion may by disturbed by altered salivary flow and composition (4). It is considered that determination of some biomarkers in saliva can be effective alternative method for monitoring the efficacy of the treatment with dialysis in CKD patients.

Our study evaluated the salivary secretion of patients with chronic renal failure by evaluating whole saliva, which is a mixed oral fluid formed by secretion of the major and minor salivary glands. Although in the whole saliva there are components of non-salivary origin, whole saliva is a good source of salivary components for evaluation.

Salivary Flow rate and pH:

In our study, assessment of the salivary flow rate before and after a dialysis session (Before - 0.4 +/- 0.2ml/min and after - 0.8 +/- 0.3 ml/min) was found to be statistically significant (*P* < 0.005). No major differences in salivary pH were found in the study sample, before and after a dialysis session. This result is consistent with the previous study by Martins *et al.* (8) who also found statistically significant difference between the salivary flow rate before and after a hemodialysis session, and also between patients on hemodialysis and healthy controls. Similar results were obtained by Kaushik *et al.* (9) who found statistically significant lower stimulated and unstimulated salivary flow rate in patients on dialysis when compared to healthy controls.

Bayraktar *et al.* (10) reported the stimulated salivary flow rate to be significantly lower in patients on hemodialysis when compared to patients on peritoneal dialysis and healthy controls. They also found a statistically significant higher salivary pH and buffer capacity in patients on peritoneal and hemodialysis when compared to controls. In a study by Al Nowaiser *et al.* (11) there was no significant difference in saliva flow rate in the CRF children compared with the controls. The buffering capacity and pH was significantly greater in the CRF group when compared with the controls.

Biochemical composition of Saliva:

Normal blood urea concentration is 30-40 mg/dl where as the normal urea of saliva is 12-70 mg/dl. The normal range of serum creatinine is 0.6 – 1.5mg/dL and salivary creatinine is 0.05–0.2 mg/dL. In the present study, we found a statistically significant difference in the concentrations of salivary urea, creatinine, potassium and chloride.

This result is consistent with the results obtained by Seethalakshmi *et al.* (12) who did a Correlation of Serum and Salivary Bio-chemical Parameters in end Stage Renal Disease Patients Undergoing Hemodialysis in Pre and Post-Dialysis State. They found a statistically significant difference between blood and salivary urea, creatinine, potassium and phosphate levels before and after a hemodialysis session. Whereas no statistically significant difference was found in blood and salivary sodium levels before and after a hemodialysis session

Serum and salivary creatinine levels were significantly higher in CKD patients than controls, in the study done by Venkatapathy *et al.* (13) they found a positive correlation between serum and salivary creatinine levels in patients on dialysis and a negative correlation in controls. The increased serum creatinine levels in CKD patients create a concentration gradient that facilitates increased diffusion of creatinine from serum into saliva. So, a good positive correlation can be seen in CKD patients.

In the study by Martins *et al.* (8) on the Salivary analysis of patients with chronic renal failure undergoing hemodialysis, a statistically significant difference was found between the salivary flow rate, concentrations of magnesium, phosphorus and potassium, whereas no significant difference was seen between salivary concentrations of sodium and calcium. They concluded that in patients with chronic renal failure, saliva composition is altered, and this may be reflected in the oral cavity. However, periodic hemodialysis seems to help control the flow and composition of saliva.

Walt *et al.* (14) reported on a series of salivary markers that were associated with end stage renal disease. The list of markers included were Nitrite, Uric acid, sodium, chloride, pH, amylase and lactoferrin. Saliva levels of Nitrite and Uric acid consistently tracked dialysis, exhibiting decreasing concentrations throughout the process; the rate of decrease, however, varied by individual. The levels of Calcium, Magnesium, Phosphate and Potassium were found to be statistically insignificant. In a subsequent study by Blicharz *et al.* (15) colorimetric test strips were used to monitor salivary nitrite and uric acid before and after hemodialysis. It was suggested that a salivary test could be used by patients to decide when dialysis is required, thereby eliminating unnecessary visits to a dialysis clinic.

In our study, we analyzed the flow rate, pH and the biochemical components of saliva such as urea, creatinine, sodium, potassium, chloride and calcium before and after a hemodialysis session, so as to determine the effects of hemodialysis on the salivary composition and also to determine the biomarkers for monitoring the effectiveness of hemodialysis. We found a statistically significant difference in the salivary flow rate and concentrations of urea, creatinine, chloride and potassium.

The present study did not consider the age related changes in salivary flow rate and composition. Total daily flow of whole saliva measures, on average, between 500 mL and 1.5 L, depending on the reference. There are daily and annual ebbs and peaks in the salivary flow. Circadian (daily) low flow occurs during sleep, whereas peaks occur during high stimulation periods. Circannual (yearly) low flow occurs during the summer, whereas peak flow is during the winter. Circadian flow variations affect not only flow but also the concentration level of salivary components such as salivary electrolytes and proteins. Several factors may influence salivary flow and its composition. As a result, these vary greatly among individuals and in the same individual under different circumstances. Changes in the flow of saliva, pH values and biochemical composition are reflected on the oral clinical finding.

Further researches considering the above mentioned salivary biomarkers may shed more light on the accuracy of the various salivary components in determining the effectiveness of dialysis and also on the effects of dialysis on salivary composition and function.
